# ERp57/PDIA3: new insight

**DOI:** 10.1186/s11658-022-00315-x

**Published:** 2022-02-02

**Authors:** Silvia Chichiarelli, Fabio Altieri, Giuliano Paglia, Elisabetta Rubini, Marco Minacori, Margherita Eufemi

**Affiliations:** 1 Department of Biochemical Sciences “A.Rossi-Fanelli”, Sapienza University of Rome, P.le A.Moro 5, 00185 Rome, Italy; 2 Enrico Ed Enrica Sovena” Foundation, Rome, Italy

**Keywords:** ERp57, PDIA3, PDI inhibitors, Punicalagin, Vitamin D3, Cancer, Infections, Nervous system, Cardiovascular system, Fertility

## Abstract

The ERp57/PDIA3 protein is a pleiotropic member of the PDIs family and, although predominantly located in the endoplasmic reticulum (ER), has indeed been found in other cellular compartments, such as the nucleus or the cell membrane. ERp57/PDIA3 is an important research target considering it can be found in various subcellular locations. This protein is involved in many different physiological and pathological processes, and our review describes new data on its functions and summarizes some ligands identified as PDIA3-specific inhibitors.

## The biological function of ERp57/PDIA3

Protein disulfide isomerases (PDIs) are an important cellular oxidoreductase enzyme family including several structurally related components. The members of this family are characterized by thioredoxin-like domains containing one or more active sites [1], with the canonical Cys–X–X–Cys sequence. Phylogenetic analysis of the human PDI family and subfamilies revealed a high correlation among the members along with an evident evolutionary divergence [2]. PDIs are involved in protein folding, catalyzing the formation and remodeling of disulfide bonds, and are mainly located in the endoplasmic reticulum (ER). Several studies have established that at least some members of this family may also be found in small quantities in different subcellular [3] and extracellular [4] locations, even though an ER retention sequence is present at the C-terminus of the protein sequences. To escape from the ER, the PDIs use different pathways, and KDEL endoplasmic reticulum protein retention receptor 1 (KDELR1) could be responsible for the transport of PDIs to the cell surface, plausibly near their target molecules [5]. Due to their conformational plasticity, PDIs display distinct substrate specificities and chaperone activities [6].

The human PDI family members display, beyond the presence of a thioredoxin-like domain, considerable differences in length and domain arrangement [2]. The first identified member of this protein family is PDIA1 (prolyl 4-hydroxylase subunit beta, *P4HB* gene), and it is structurally characterized by two thioredoxin-like active domains (a, a′), two substrate-binding domains (b, b′) with a hydrophobic pocket in the b′ domain, a linker sequence between the b′ and the a′ domains, and a C-terminal extended domain. The two PDI members, PDIA1 and ERp57/PDIA3, have considerable overlap in their whole protein structure, but their differences are due to their different evidenced cellular biochemical roles in cellular homeostasis. For instance, PDIA1 catalyzes oxidative folding of non-glycoproteins via binding to the hydrophobic pocket of the b′ domain, while ERp57/PDIA3 promotes oxidative folding of glycoproteins selectively in concert with lectin chaperones calnexin and calreticulin [2, 6, 7]. Moreover, their enzymatic activity varies due to their differing redox potentials [8].

The ERp57/PDIA3 protein is a pleiotropic member of the PDIs family, also known as P58, ER60, ERp57, ERp60, ERp61, GRP57, GRP58, PI-PLC, HsT17083, HEL-S-269, HEL-S-93n, and 1,25D3-MARRS (https://www.ncbi.nlm.nih.gov/gene/2923), which henceforth we will call ERp57/PDIA3. Of note, PDIA3 was erroneously named phospholipase C alpha when first sequenced [9], attributing a phospholipase activity to it, while the name GRP58 was attributed from the evidence that PDIA3 increases after glucose depletion [10] and the name 1,25D3-MARRS is linked to the discovery that this protein is likewise a cell membrane receptor of 1α,25(OH)_2_D_3_ (vitamin D_3_) associated with the rapid actions of vitamin D [11, 12]. The ERp57/PDIA3 has a noncanonical ER retention signal (QEDL) on the C-terminal domain and a Lys-rich nuclear localization signal, which binds with high affinity to a specific site of importin, responsible for the nuclear import process [13]. ERp57/PDIA3, found in different extracellular and subcellular locations, is involved in multiple processes [14]. To date, specific transcriptional or post-transcriptional regulators of ERp57/PDIA3 are not well known, but a study, conducted with high-throughput sequencing of small RNA libraries (from liver tissues of rainbow trout specimens), identified and characterized some miRNAs that are probably important in the response to heat stress, and three of them (let-7b, let-7c, and miR-145) appeared to downregulate ERp57/PDIA3 [15].

ERp57/PDIA3 is characterized by four domains, called respectively a, b, b′, and a′, each one with a thioredoxin-like fold [1, 16]. The first and the fourth domain (a and a′ domains), carry a thioredoxin-like active site, with a C–G–H–C sequence, and are responsible for ERp57/PDIA3 redox properties [17]. The b and b′ domains are necessary for the full activity of the protein and provide the binding sites for calreticulin or its membrane-bound homologous calnexin. The binding of glycoproteins to the calreticulin–PDIA3 complex is necessary for the catalytic redox activity of PDIA3. The interaction of calreticulin and ERp57/PDIA3, important for the assembling of the human MHC-I peptide-loading complex, has recently been well studied with low-resolution cryo-electron microscopy (cryo-EM) [18]. Moreover, ERp57/PDIA3 is involved in the redox activation of protein kinase RNA-like ER kinase (PERK) in response to protein unfolding [19]. This involvement is particularly interesting considering the role of ER stress and unfolded protein response (UPR) in cardiovascular diseases as described in a review by Ren et al. The review summarizes the genes involved in ER functions, ER stress, or the UPR that (modified in mouse models) often provides pathological cardiovascular phenotypes [20]. Moreover, other roles and functions of ERp57/PDIA3 complexed with calreticulin and calnexin [21, 22] are well described in a recent review by Lam and Lim [23].

Recently, a reflux of proteins from the ER to the cytosol was analyzed by Sicari et al. [24]. They found that this reflux mechanism is conserved in mammalian cells and in cancer cells (i.e., human and murine cells). Moreover, this process is constitutively activated in cancer cells, where refluxed ER proteins play a gain-of-function role in the cytosol, acting as inhibitors of tumor suppressor. Among refluxed ER proteins, the cytosolic fraction of ERp57/PDIA3 in cancer cells is enriched up to ∼ 70% compared with only ∼ 10% enrichment in non-cancer control cells.

## Localization and functions of ERp57/PDIA3 outside of the ER

Although ERp57/PDIA3 is predominantly located in the ER, it has indeed been found in other cellular compartments, such as the nucleus and the cell membrane. The release of ERp57/PDIA3 from the ER was first observed by Hirano and collaborators [25], who noticed that the protein was being secreted from 3T3 cells.

### Nucleus

The nuclear localization of ERp57/PDIA3 was discovered many years ago [3, 26] and is not unusual; nevertheless, the role of the protein in the nuclear compartment is not well understood. It has subsequently emerged that PDIA3 participates in the signal transduction processes of STAT3 pathways [27, 28]. Sehgal et al. found ERp57/PDIA3 in the lipid raft fraction of the cell membrane associated with STAT3 [29] (a member of the family of signaling and transcription factors 3 with a significant role in inflammation and deregulated in numerous tumors) and caveolin-1 [30]. Various studies conducted on cancer cells have shown ERp57/PDIA3 involvement in signal transduction of STAT3 [26, 28, 31–33]. A study on clear cell renal cell carcinoma (ccRCC) highlighted the increased levels of ERp57/PDIA3 and interleukin enhancer-binding factor 3 (ILF3) in ccRCC tissue, which were correlated with poor patient survival. The research article found evidence that ILF3 expression is transcriptionally regulated by a ERp57/PDIA3–STAT3 complex and that ILF3 may bind to the 3′ UTR of ERp57/PDIA3 mRNA to positively regulate ERp57/PDIA3 expression by enhancing its mRNA stability. These data indicate a PDIA3/STAT3/ILF3 feedback loop with a key role in the proliferation mechanism of ccRCC, providing a potential target for therapeutic treatment [31]. Moreover, another study on hepatocellular carcinoma (HCC), where the increased expression of ERp57/PDIA3 was a marker of poor prognosis, revealed a specific role of ERp57/PDIA3 in association with phosphorylated (pY705) STAT3 on HCC progression. In particular, the research suggested that ERp57/PDIA3 contributes to the aggressive phenotype of HCC through its association with the STAT3 signaling pathway by the regulation of STAT3 transcriptional potential [34].

In HepG2 (hepatocellular carcinoma) cancer cells, ERp57/PDIA3 undergoes a nuclear translocation after tumor necrosis factor-α treatment [35]. It may also cooperate with Ref-1/APE in the reductive activation of transcription factors [36], and in this regard the flavonoid silibinin appears to enhance the formation of a ERp57/PDIA3–Ref-1 complex and affect its intracellular distribution [37]. Our group showed a specific interaction of ERp57/PDIA3 with several different DNA sequences in HeLa (cervix adenocarcinoma) [38] and melanoma [39] cell lines. Jung et al. associated the ERp57/PDIA3 protein with the expression of dyskerin pseudouridine synthase 1 (DKC1), a RNA-binding protein and an essential component of the telomerase holoenzyme. In HCC cells, the authors identified ERp57/PDIA3 as a repressor of DKC1 by recognizing a G-quadruplex structure at the DKC1 locus. Moreover, they observed that oxidative modification of ERp57/PDIA3 at the level of Met338 residue caused its redistribution to the cytosol. Therefore, reactive oxygen species (ROS) levels affected ERp57/PDIA3 cytosolic levels, and this can result in HCC cell survival through DKC1 upregulation [40].

### Mitochondrion

ERp57/PDIA3 is present in mitochondria-associated membranes (MAM), the regions of ER that are closely associated with mitochondria [41, 42]. ERp57/PDIA3 has also been found in the mitochondrial compartment complexed with μ-calpain, and this complex is able to cleave apoptosis-inducing factor (AIF), a protein that in the truncated form is involved in the DNA fragmentation process in programmed cell death [43]. ERp57/PDIA3 and PDIA1 seem to possess a similar pro-apoptotic function by inducing Bak oligomerization [44]. Furthermore, in HeLa cells, ERp57/PDIA3 could be implicated in mitochondrial Ca^2+^ uptake due to an involvement in the expression of the mitochondrial Ca^2+^ uniporter (MCU) [45]. ERp57/PDIA3 could also be involved in the regulation of mitochondrial Ca^2+^ indirectly through its interaction with calnexin. In particular, palmitoylated calnexin interacts with sarcoendoplasmic reticulum (SR) Ca^2+^ transport ATPase (SERCA) 2b, and this complex acts on ER Ca^2+^ content and regulates ER–mitochondria Ca^2+^ crosstalk. In this respect, ERp57/PDIA3 interacts with nonpalmitoylated calnexin in the ER in the protein quality control system [46]. A more recent study revealed a novel role for ERp57/PDIA3 in suppressing mitochondrial bioenergetic function via the inhibition of STAT3 Ser727 phosphorylation. In particular, the authors investigated the role of ERp57/PDIA3 in immortalized human cerebral microvascular cells (CMECs). They analyzed mitochondrial bioenergetic function in CMEC wild-type cells and ERp57/PDIA3 silenced ones, where the gene was deleted using CRISPR–Cas9. The ERp57/PDIA3 deletion suppressed mitochondrial bioenergetic function and caused an increase in the phosphorylation level of STAT3 S727 residue, which is known to promote this function. Moreover, in ERp57/PDIA3^−/−^ CMECs, the mitochondrial membrane potential and reactive oxygen species production was increased, but not mitochondrial mass. Finally, ERp57/PDIA3^−/−^ CMECs were more resistant to oxygen–glucose deprivation [47].

### Cell surface

To date, several observations have indicated the cell surface location of ERp57/PDIA3 through the interaction with cell membrane proteins, or even its extracellular localization. Several years ago, the ERp57/PDIA3 was erroneously identified as phospholipase C alpha and as interacting with vasopressin [48] and angiotensin II [49] receptors. Subsequently, ERp57/PDIA3 was detected on the cell surface in complex with calreticulin, and their localization was correlated to “immunogenic cell death” (ICD). This is a peculiar type of apoptosis where apoptotic cells can be detected by the immune system and elicit an adaptive immune response specific to dead-cell-associated antigens [50, 51]. In particular, ERp57/PDIA3 has been shown to be necessary for the translocation of calreticulin to the plasma membrane [52]. More recently, a study on Jurkat (acute T-cell leukemia) cells showed a role of integrins and ERp57/PDIA3 in the regulation of cell surface translocation of calreticulin; in particular, the authors hypothesized the involvement of plasma membrane integrins as inhibitors and ERp57/PDIA3 as promoter of calreticulin translocation [53]. A study conducted on a xenograft model, where MDA-MB-468 (breast adenocarcinoma) cells were injected into female Balb/c-nude mice, showed that EGFR phosphorylation in MDA-MB-468 cells is inhibited after treatment with silibinin, a PDIA2 ligand [54]; it is possible that this event is related to the role of ERp57/PDIA3 in EGFR signaling and internalization, as evidenced by silencing of ERp57/PDIA3 in MDA-MB-468 cells [55]. Fujii et al. also found ERp57/PDIA3 abundantly expressed in the apical canalicular membrane of gastric parietal cells, where it modulates the activity of H^+ ^/K^+^-ATPase, suggesting a novel function of ERp57/PDIA3 in addition to its chaperoning function [56].

ERp57/PDIA3 was also identified as a membrane-associated receptor (1,25D3-MARRS) for 1α,25(OH)2D3 (1α,25-dihydroxyvitamin D_3_), the biologically active form of vitamin D_3_. Its function is associated with the rapid and nongenomic response to the hormone [12, 57, 58]. ERp57/PDIA3 was identified as an alternative cell membrane receptor for active forms of vitamin D_3_ that regulates some phenotypic functions [59, 60]. Boyan et al. studied the specific role of ERp57/PDIA3, vitamin D receptor (VDR), and caveolin-1 in the multiple signaling pathway activated by 1α,25(OH)2D3 as genomic and nongenomic response. ERp57/PDIA3 and VDR, separately, can form complexes with caveolin-1 and can activate their own downstream mediators such as phospholipase A2-activating protein (PLAA) and Rous sarcoma oncogene (c-Src), respectively. These complexes respond to 1α,25(OH)2D3 by translocation and changes in protein–protein interaction. The data demonstrate the importance of both receptors and their interactions in mediating both rapid and genomic responses to 1α,25(OH)2D3 [61]**.** The rapid action of 1α,25(OH)2D3 involves the ERp57/PDIA3–PLAA complex and requires Ca^2+^/calmodulin-dependent protein kinase II (CaMKII) in order to act on PLA2. [62]. The arachidonic acid released by PLA2 acts directly and indirectly to stimulate protein kinase C (PKC) [63]. ERp57/PDIA3 plays an important role in mediating the effects of 1α,25(OH)2D3 and bone morphogenetic protein-2 (BMP2) on mineralization, suggesting a possible fundamental role in mediating extracellular matrix mineralization [64]. Rapid activation of Wnt family member 5A (WNT5A) by 1α,25(OH)2D3 was shown to be also dependent on the ERp57/PDIA3 membrane complex [65]. Boyan et al. suggested a regulation of endochondral bone formation, via a rapid response to steroid hormones mediated by extracellular matrix vesicles. This mechanism involves the interaction of 1α,25(OH)2D3 with VDR and ERp57/PDIA3 via genomic and rapid membrane-associated signaling pathways [66]. In summary, the different functions of vitamin D_3_ involve, quite often, its interaction with ERp57/PDIA3. Vitamin D_3_’s effects on prostate cancer biology were highlighted in a review underlining that adequate levels of vitamin D_3_ and androgens slow or halt prostate cancer progression, particularly for patients with early-stage and locally confined disease [67]. It has been demonstrated that 1α,25(OH)2D3 is able to mediate both genomic and nongenomic responses (through its interaction with ERp57/PDIA3) in prostate cancer (CaP) cells, and it has been suggested as an important therapeutic agent in inhibiting prostate cancer progression [68]. In this respect, Larsson et al. demonstrated that 1α,25(OH)2D3–PDIA3 interaction decreases invasiveness of LNCaP (prostate cancer) cells with the involvement of the JNK/SAPK MAPK signaling pathway [69]. More recently, Diaz Cruz et al. evaluated the expression of VDR and ERp57/PDIA3 in different prostate cancer (PCa) cell lines (PNT2, P4E6, LNCaP, DU145, and PC3), representing different stages of tumor progression, and identified a novel transcript isoform of ERp57/PDIA3 (PDIA3N, an N-truncated isoform of ERp57/PDIA3; ENST00000538521.1, GRCh37.p13 Ensembl 2018). This study showed that the mRNA expression ratio PDIA3N:PDIA3 is higher in androgen-independent cell lines than in androgen-dependent cell lines [70]. The role of vitamin D_3_ in brain physiology [71] and neurological disease [72, 73] has been studied. Landel et al. observed different expression levels of VDR and ERp57/PDIA3 in several tissues of Wistar rats. VDR appears less abundant in brain tissue, whereas ERp57/PDIA3 is highly expressed; moreover, VDR is expressed mostly in astrocytes, while ERp57/PDIA3 is present in every brain cell type. The authors suggested that, in different brain cell types, ERp57/PDIA3 is probably the only regulator of vitamin D_3_ actions [74]. Dursun et al. observed that silenced neurons (both SiVDR and SiERp57/PDIA3/1,25MARRS or double silenced) showed intracellular Aβ1–42 production. However, treatment with 1α,25(OH)2D3 attenuated Aβ1–42 intracellular production and secretion. The authors suggested a role of ERp57/PDIA3 in loss of control over APP processing in the ER or in the early secretory pathway, but it is unclear whether this process involves vitamin D_3_ [75]. In a short review, Gezen-Ak and Dursun described 1α,25(OH)2D3 as a “neurosteroid” and not a simple vitamin; they underlined that its involvement in neurodegeneration mechanisms is complex and not yet elucidated, so a more in-depth study is needed [76]. Compelling research has delved into the administration of vitamin D_3_ against *Helicobacter pylori* (*H. pylori*) infection. The authors used C57BL/6 mice as a model and extended the research to a human normal gastric epithelial cell line (HFE145 cells). During infection, *H. pylori* was sequestered into the gastric epithelium and survived in autophagosomes with impaired lysosomal acidification. Vitamin D_3_ administration was able to restore the lysosomal degradation function by activating its rapid response receptor ERp57/PDIA3, which translocates into the nuclear compartment as a ERp57/PDIA3–STAT3 protein complex and then upregulates the mucolipin 3 (MCOLN3) channels. This results in the enhancement of Ca^2+^ release from lysosomes and normalization of lysosomal acidification that leads to *H. pylori* elimination through the autolysosomal pathway [77]. More recently, Zmijewski and Carlberg argued, in an excellent review, over the rapid and nongenomic pathways and the possible role of 1α,25(OH)2D3 in a meaningful addition to the genome-wide effects. The conclusions of the “dispute” indicated VDR as the receptor of choice in 1α,25(OH)2D3 signaling; nevertheless, the authors pointed out that ERp57/PDIA3, involved in a spectrum of intracellular rapid and nongenomic responses, could be of help to explain the activity of low- or non-calcemic analogs of vitamin D_3_ [78].

ERp57/PDIA3, with a noncanonical ER localization, was also detected on the surface of the sperm head, and it was demonstrated to be required for sperm–egg fusion. A research study by Ellerman et al. showed that antibodies against this protein blocked sperm–egg fusion, and it was hypothesized that ERp57/PDIA3 can remodel integrins of sperm surface [79]. Afterwards, ERp57/PDIA3 was found to be downregulated in males with low fertilization rates [80, 81]. In particular, Zhang et al. found that ERp57/PDIA3 expression was downregulated in a cohort of male in vitro fertilization (IVF) patients with low fertilization rates, and they proposed ERp57/PDIA3 as a novel phenotype marker for male infertility [80]. In mice, ERp57/PDIA3 was demonstrated to have a function in sperm capacitation. ERp57/PDIA3 was found upregulated in irradiated testis, suggesting that this chaperone has a protective effect on the testis, maintaining normal spermatogenesis when exposed to radiation [82]. Moreover, ERp57/PDIA3 is likely to be related to the thiol–disulfide exchange reactions for the gamete fusion process. ERp57/PDIA3 was identified as a component of the human spermatozoa–ZP receptor complex in which ZP3 (a glycoprotein among the four that participate in the ZP receptor complex) is the primary binding site of acrosome reaction of human spermatozoa receptors [79]. The data suggested the translocation of ERp57/PDIA3 to the sperm surface in the acrosomal region during capacitation, and showed that capacitated spermatozoa treated with a PDI inhibitor had significantly lower ZP-binding capacity [80]. Considering that ERp57/PDIA3 is a thiol oxidoreductase, another study proposed that changes in the surface thiol status can alter the ZP-binding capacity of human spermatozoa [83].

Regarding ERp57/PDIA3 located on the cell surface, Wu et al. hypothesized that ERp57/PDIA3 present on the platelet surface mediates platelet aggregation and is also required for hemostasis and thrombosis [79, 80, 84]. In this context, ERp57/PDIA3 appears to have a specific role in the activation of the αIIbβ3 integrin (platelet fibrinogen receptor) and is required for the incorporation of platelets into a growing thrombus [85]. The evidence on the role of ERp57/PDIA3 in the modulation of αIIbβ3 integrin affinity suggests that the selective inhibition of extracellular thiol isomerases may enable the development of new antithrombotic drugs [86]. Different PDIs (e.g., PDIA1, ERp57/PDIA3, ERp5, and ERp72) were identified as positive regulators of platelet function and thrombosis [87], and thioredoxin-related transmembrane protein 1 (TMX1) was described as oxidizing the αIIbβ3 integrin thiols, acting as a negative regulator of platelet aggregation [88]. ERp57/PDIA3 regulates the lectin pathway of complement activation by cleaving disulfide bonds in ficolin-3 (an abundant lectin initiator of the complement pathway in humans through its disulfide-linked multimers) and reducing its multimer size [89]. The thiol isomerase inhibitors were suggested as a new class of antithrombotic agents; the development of inhibitors that are unable to gain access to intracellular thiol isomerases may be challenging, given their vital and ubiquitous roles in protein folding [90, 91]. A recent study found that ERp57/PDIA3 colocalized with extracellular human transglutaminase 2 (TG2) in cultured human umbilical vein endothelial cells (HUVECs) [92]. TG2 protein is often dormant in the extracellular matrix under normal physiological conditions and is activated under specific conditions such as tissue injury or inflammation, with its activation being specifically operated by extracellular thioredoxin-1 (Trx) [93]. Yi et al. showed that ERp57/PDIA3 oxidatively inactivates human transglutaminase 2, thus defining a reversible protein-controlled redox switch system [92]. Two recent reviews analyzed the role of the thiol isomerase “system” as positive and negative regulators that contribute to redox homeostasis, maintenance of normal hemostasis, vascular integrity [94], and regulation of thrombotic events [95].

## ERp57/PDIA3 and diseases

### Neurological disease

Various studies have identified ERp57/PDIA3 in the nervous system, and several research works have provided evidence for its involvement in neurodegeneration [96]. The role of ERp57/PDIA3 was linked to ER stress pathways [97], which characterize diseases such as Alzheimer’s disease (AD) [98] and amyotrophic lateral sclerosis (ALS) [99] (in this case, ERp57/PDIA3 also plays a role because of its interaction with the MAM compartment [100]). At the same time, it was hypothesized that ERp57/PDIA3 involvement in neurodegeneration diseases is related to its role as vitamin D_3_ receptor [76]. A protective function of ERp57/PDIA3 in nervous system diseases was suggested by using a Tat-ERp57/PDIA3 fusion protein (where the Tat domain was used to deliver the protein without any cellular toxicity). This study showed that Tat-ERp57/PDIA3 acts as a neuroprotective agent against ischemia by attenuating oxidative damage and blocking the apoptotic pathway related to the UPR [101]. Another study identified ERp57/PDIA3 as protective against mutant SOD1-induced cellular pathology in amyotrophic lateral sclerosis. The authors analyzed the role of ERp57/PDIA3, first in a neuronal cell line (co-expressing superoxide dismutase 1 and ERp57/PDIA3) and then in primary cortical neurons. The obtained results showed that overexpression of ERp57/PDIA3 inhibited mutant SOD1 inclusion formation, ER stress, and apoptosis, whereas silencing of ERp57/PDIA3 expression enhanced mutant SOD1 inclusion formation, ER stress, and toxicity, indicating a protective role for ERp57 against SOD1 misfolding. Moreover, the authors partially colocalized ERp57/PDIA3 with TAR DNA-binding protein-43 (TDP-43)-positive inclusions in spinal cords from sporadic ALS patients, then linking ERp57/PDIA3 to protein misfolding events in human sporadic disease [102]. A recent research, using mutant superoxide dismutase 1 (SOD1) mice as an experimental ALS model, provided evidence that ERp57/PDIA3 protects early muscle denervation. The study suggested that the protein acts at early stages by sustaining motoneuron connectivity through the interference of SOD1 aggregation probably via mixed disulfide crosslinks [103]. Nevertheless, a study by Wang et al. on traumatic brain injury (TBI) showed that ERp57/PDIA3 deficiency reduces inflammation in brain samples of mice after TBI and ERp57/PDIA3 knockout attenuates inflammation, oxidative stress, and apoptosis in oxygen–glucose deprivation and re-oxygenation (OGDR)-treated astrocytes [104]. ERp57/PDIA3’s role in prion infection as a cellular factor controlling prion protein biosynthesis also deserves mention [105]. In fact, Thapa et al. showed that ERp57/PDIA3-overexpressing cells significantly reduced ER stress, accompanied by a decrease in prion protein (PrP) aggregates, and lentiviral ERp57/PDIA3 expression prolonged the survival of prion-infected mice [106].

### Cancer

The role of ERp57/PDIA3 in cancer was shown years ago, and its involvement in cancer progression suggests a potential use of ERp57/PDIA3 both as a marker and a therapeutic target [2]. A recent review summarized the current knowledge on ERp57/PDIA3 in cancer and the mechanisms by which this protein is involved, starting from carcinogenesis and extending to its potentialities as a therapeutic target. The authors have provided an overview of the role of ERp57/PDIA3 in the regulation of T cell-mediated immune responses, immunogenic cell death, UPR, DNA repair signaling, and membrane-initiated signaling pathways [107]. An interesting recent study analyzed the role of ERp57/PDIA3 by the inhibition of its activity, with a small molecule known as 16F16. This treatment destabilizes mechanistic target of rapamycin complex 1 (mTORC1) and enhances the effect of the mTOR inhibitor everolimus (Ev), increasing cultured liver cancer cells’ susceptibility to Ev. The inhibition was related to the interaction of ERp57/PDIA3 with mTOR and the reduction in the phosphorylation level of eukaryotic translation initiation factor 4E -binding protein 1 (4E‑BP1) [108]. Another study investigated the role of ERp57/PDIA3 in an aggressive cancer such as glioblastoma (GB) tumor and its crosstalk with microglia. The study was performed on GB cells and glioma-associated microglia/macrophages (GAMs) collected from patients’ specimens after surgical tumor resection. After gene silencing or specific inhibition of ERp57/PDIA3 by punicalagin, the authors demonstrated that ERp57/PDIA3 is involved in the pro-tumor activity of GB cells and GAMs. The ERp57/PDIA3 silencing seems to activate GB cells to produce IL24 (a tumor-suppressing protein) and leukemia inhibitory factor (LIF), inhibiting cell differentiation. The reduced PDIA3 expression/activity in GB cells significantly limited the microglia pro-tumor polarization towards the M2 phenotype and the production of pro-inflammatory factors, supporting a role of PDIA3 expression in GB-mediated pro-tumor activation of microglia. However, ERp57/PDIA3 activity in gliomas is still poorly characterized and could be related to induction of autophagic cell death and apoptosis [109].

Another study identified ERp57/PDIA3 as being indirectly involved, with a mutant calreticulin gene (CALR), in altered Ca^2+^ mobilization in patients with myeloproliferative neoplasms. A somatic mutation of CALR results in the dissociation of the ERp57-mediated complex between calreticulin and stromal interaction molecule 1 (STIM1, protein of the store-operated calcium entry machinery) leading to Ca^2+^ mobilization [110]. These results correlated with the findings that mutant CALR constitutively activates the thrombopoietin receptor (myeloproliferative leukemia protein, MPL), even in the absence of thrombopoietin, thus inducing cellular transformation [111]. Moreover, Pronier et al. analyzed the CALR interactome in myeloproliferative neoplasms and demonstrated that the ERp57/PDIA3–CALR complex binds to the MPL promoter, regulating its expression, in CALR-mutant cells. They observed a lower expression of MPL mRNA in control cells (wild-type CARL) even if only ERp57/PDIA3 was silenced, suggesting that ERp57/PDIA3 itself has a regulatory function of the locus [112].

### Infections disease

The pleiotropic functions of PDIs in general and of ERp57/PDIA3 in particular include their role in infections disease. ERp57/PDIA3 was shown to be directly involved in influenza virus replication [113]; Kim and Chang conducted a knockdown study using small interfering RNAs (siRNAs) for ERp57/PDIA3 and other PDIs (PDI1 and PDIA4). The knockdown of ERp57/PDIA3 led to a reduction of influenza A and B virus replication in Madin–Darby canine kidney (MDCK) and A549 (human lung cell line) cells, indicating that this protein plays an important role in influenza virus replication and that it acts after virus entry [114]. A study on ERp57/PDIA3 expressed in mouse lung epithelial cells suggested the importance of this protein in the formation of disulfide bonds of influenza A virus (IAV) hemagglutinin (HA) and the subsequent establishment of infection, inflammation, and airway hyperresponsiveness (AHR) [115]. A more recent study, conducted both in vitro and in vivo, further investigated the role of ERp57/PDIA3 in H3N2 virus HA stabilization, and the results obtained suggest that ERp57/PDIA3 could improve the stability and immunogenicity of hemagglutinin [116]. A study on nitazoxanide and its circulating-metabolite tizoxanide, clinically used as an antiprotozoal/antimicrobial drug and more recently as an antiviral against hepatitis-C virus, rotavirus, and influenza viruses, suggested that its broad-spectrum antiviral activity could depend on the inhibition of ERp57/PDIA3 activity, because of the role of this protein in the correct disulfide-bond architecture of viral glycoproteins [117]. Moreover, a recent review by Santoro et al., regarding the severe acute respiratory syndrome coronavirus 2 (SARS-CoV-2) spike glycoprotein, suggested that ERp57/PDIA3 may be involved in the biogenesis, correct folding, and maturation of the SARS-CoV-2 spike protein [118]. The role of UPR and protein disulfide isomerases in pulmonary viral infection and disease was well summarized in a recent review of Chamberlain and Anathy [119]. More recently, a review was published on the role of ERp57/PDIA3 in viral infections, emphasizing that the presence of this protein on the cell surface assists different viruses to enter the cells and replicate [120].

## High-impact information on ERp57/PDIA3 (ligand)

ERp57/PDIA3 is an important research target considering it can be found in various subcellular locations. Examples are provided below such as its involvement in platelets aggregation, sperm–egg fusion, and vitamin D_3_ signaling due to its membrane localization.

Evidence has been provided for the role of ERp57/PDIA3 in platelet aggregation, and the study of potential inhibitors of the redox activity of ERp57/PDIA3 could be interesting to identify promising candidates for antithrombotic therapy targeting ERp57/PDIA3 [90, 121, 122]. Years ago, a study on the antithrombotic activity of red wine and red grape juice showed that natural components of wine act as PDI inhibitors and, to a lesser extent, as ERp57/PDIA3 inhibitors [123]. More recently, Gaspar and Gibbins analyzed the function of thiol isomerases in thrombosis and hemostasis and summarized the known PDI inhibitors, showing that often these inhibitors are not so selective for a specific PDI family member [95].

The study of ERp57/PDIA3’s role in human fertility would allow this information to be used for two antithetic but equally interesting purposes. First, the mechanism of molecular action of the protein expressed in spermatozoa could be discerned, using targeted inhibitors. Second, specific inhibitors could be considered for a possible contraceptive function. The function of ERp57/PDIA3 in sperm capacitation can be hypothesized to be related to its interaction with vitamin D_3_ [124, 125]. In fact, it should pointed out that several functions of spermatozoa, such as viability, integrity of DNA, motility, capacitation, acrosome reaction, and fertilizing capability, are targets of the action of hormones, including estrogens and 1α,25(OH)2D3 [126].

Besides, cumulative evidence highlights the impact of vitamin D_3_ deficiency as a favoring factor in various neurological diseases, and different studies focused on determining the beneficial effect resulting from its supplementation (see review [73]). Potential inhibitors of the interaction between vitamin D_3_ and ERp57/PDIA3 could be used to discriminate their role in neurological disease. Moreover, it was shown that 1α,25(OH)2D3 can induce autocrine or paracrine rapid nongenomic actions via ERp57/PDIA3, whose transcript is abundantly expressed in all cerebral cell types [74].

Deciphering the actions of ERp57/PDIA3 as an extracellular disulfide isomerase or as a receptor of vitamin D_3_ will help us obtain knowledge on its role in promoting health.

Recent reviews have summarized the different PDIs inhibitors currently known [2, 95, 127]. However, to date, not many specific ERp57/PDIA3 inhibitors have been identified (Table 1), and in fact some of them are pan-style inhibitors for the PDI family members.

**Table 1:** ERp57/PDIA3 inhibitors

Inhibitor	Effect	IC50 (Kd)	Binding site	References
Vancomycin	Inhibition reductase activity	> 100 μM (Kd 6.7 μM)	ND	[17, 128]
Neomycin	Inhibition reductase activity	> 100 μM (Kd 20 μM)	ND	[17]
ADTM (derivative from Danshen, *Salvia miltiorrhiza*)	Inhibition redox activity (antiplatelet aggregation)	100–300 μM	Hydrogen bonds with Arg47, Ile453, and Phe450 a′ domain	[121, 122]
Rosmarinic acid (from Danshen, *Salvia miltiorrhiza*)	Inhibition redox activity (antiplatelet aggregation)	176.82 μM	Hydrogen bonds with Ser312, Lys366, Asp440, and Val441 a′ domain	[129]
Epigallocatechin gallate (EGCG)	Inhibition redox activity DNA-binding activity	≫ 20 μM 20 μM	Bind to the oxidized enzyme close to the active site	[131]
Silibinin	ERp57/PDIA3/REF-1 complex activator	(Kd_ox_ 0.3 μM) (Kd_red_ 0.3 μM)	Bind to the enzyme a domain or a′ domain	[37, 54, 139]
Eupatorin-5-methyl ether	Inhibition of redox activity	> 20 μM (Kd 11.1 μM)	ND	[132]
Eupatorin	Inhibition of redox activity	> 20 μM (Kd 10.3 μM)	ND	[132]
Punicalagin	Inhibition of redox activity	1.5 μM	Bind to the hydrophobic pocket on a′ domain	[109, 133–135]
LOC14	Inhibition of redox activity	4.97 μM	Reacts and oxidizes catalytic domains	[2, 115]
Dihydrotanshinone I DHT (from Danshen, *Salvia miltiorrhiza*)	Inhibition of redox activity and ERp57/PDIA3 expression	5.51 μM	Hydrogen bond between the carbonyl group and amino acid Lys433	[138]

*ND* not detected; *Kd*_*ox*_ Kd ERp57/PDIA3 oxidized; *Kd*_*red*_ Kd ERp57/PDIA3 reduced

The antibiotics vancomycin and neomycin were found to inhibit the reductase activity of ERp57/PDIA3 [17], and inhibitory activity of the antibiotic vancomycin on the ERp57/PDIA3–calreticulin interaction has been observed [128]. Cui et al. showed that ADTM, a molecule synthesized by conjugating two well-known compounds, danshensu (DSS, from danshen, *Salvia miltiorrhizae*) and tetramethylpyrazine (TMP), displayed potent inhibitory effects on the redox activity of ERp57/PDIA3. In particular, ADTM, which has a strong cardioprotective effects in vitro and in vivo, shows additionally an inhibitory effect on platelet aggregation in vitro and in vivo [121]. A structure-based virtual screening identified rosmarinic acid from *Salvia miltiorrhizae* as a major active compound against ERp57/PDIA3 [129]. A neuroprotective lead optimized compound (LOC)14 [130] inhibits specifically the ERp57/PDIA3 activity in lung epithelial cells, decreasing the intramolecular disulfide bonds and the oligomerization of HA in influenza A virus infected lung epithelial cells [115].

It was also observed the ERp57/PDIA3 has a high affinity to galloylated catechins (polyphenol of green tea), which bind closely to the thioredoxin-like redox-sensitive active sites, and a moderate inhibition of the reductase activity of ERp57/PDIA3 was observed [131]. Moreover, several other flavonoid compounds were studied, and even if the enzymatic reductase activity was almost unaffected by silibinin [37], both eupatorin-5-methyl ether and eupatorin (secondary metabolites present in fruits and vegetables) were able to inhibit reductase activity of ERp57/PDIA3 [132]. Recently, the bioactivity of extracts from whole fruit, peels, and juices of pomegranate were tested for the effect on the redox activity of ERp57/PDIA3. The obtained results demonstrate that different ratios between punicalagin [133] and ellagic acid (compounds present in fruit extracts) modulate the enzyme activity, and other ellagitannins could interfere with this inhibitory activity [134]. More recently, the specific interaction between punicalagin and ERp57/PDIA3 was analyzed by biochemical and computational approaches to identify putative binding sites and provide insights to develop useful punicalagin-based ligands [135]. In this regard, as previously reported, a study conducted on glioblastoma (GB) cells and glioma-associated microglia/macrophages (GAMs), where ERp57/PDIA3 levels are higher than in the microglia of in the surrounding parenchyma, showed that the ERp57/PDIA3 knockdown in GB cells induces different responses in GAMs and the inhibition of ERp57/PDIA3 could exert beneficial therapeutic effects. In particular, the treatment of microglial cells with the ERp57/PDIA3 inhibitor punicalagin increases apoptosis and reduces pro-inflammatory activity [109].

A clinical trial with the oral PDI inhibitor isoquercetin (also known as isoquercitrin) significantly decreased markers of coagulation in patients at risk for thrombosis [94, 136]. Moreover, different PDI inhibitors such as 16F16, PACMA31, isoquercetin, epigallocatechin-3-gallate, and nitazoxanide significantly reduced the replication of influenza A and B viruses in MDCK and A549 cells. These results suggest that PDIs are potential targets for the development of new anti-influenza drugs [114]. Recently, a cyclic peptide was identified (CSSTRESAC) that specifically acts on ERp57/PDIA3, as 1α,25(OH)2D3 receptor, through vitamin D_3_ binding protein (DBP), present on the cell surface of tumor-associated macrophages (TAM). In this study, the administration of CSSTRESAC to triple-negative breast cancer (TNBC)-bearing mice changed the cytokine profile in the direction of an antitumor immune response and delayed tumor growth [137]. The dihydrotanshinone I (from Danshen, *Salvia miltiorrhiza*) was recently identified as a potential ERp57/PDIA3 inhibitor with an anti-breast cancer effect through the UPR pathway [138].

## Conclusions

**Fig. 1:**
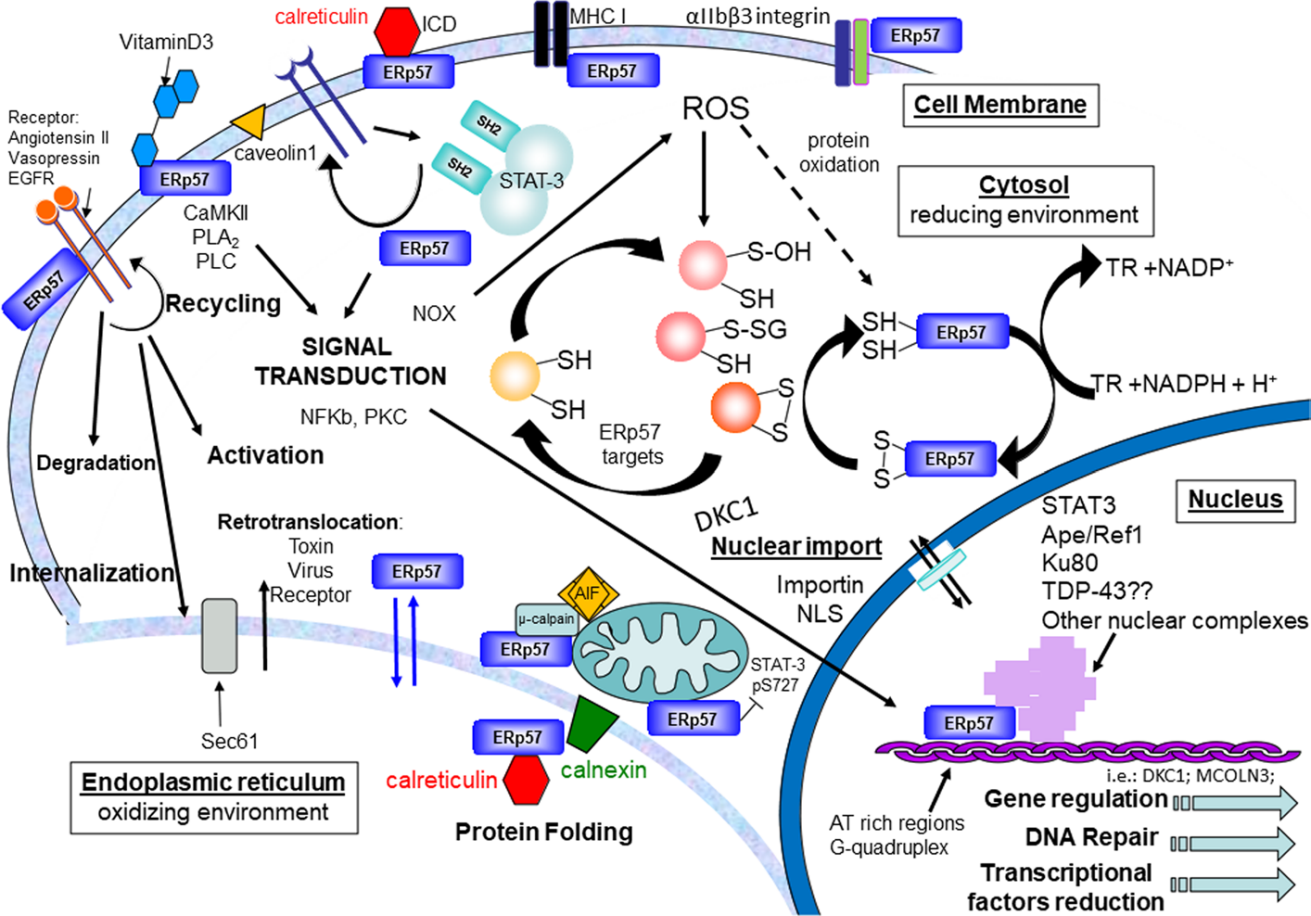
Schematic representation of the different roles of ERp57 / PDIA3 in cellular processes in different cell compartments. Cell membrane: ERp57/PDIA3 participates in assembling of MHC I, interacts with different receptors (e.g., angiotensin II, vasopressin, and EGF receptors), acts as receptor of vitamin D_3_, participates in ICD in complex with calreticulin, and interacts with αIIbβ3 integrin in platelet cells. ERp57/PDIA3 participates in different cellular processes, including signal transduction, recycling, degradation, and internalization. Cytosol: ERp57/PDIA3 interacts with STAT3 and contributes to redox balance acting on thioredoxin (TR) and other targets. Nucleus: ERp57/PDIA3 interacts with different proteins (e.g., STAT3; Ape/Ref1, Ku80, and maybe TDP-43) and different DNA sequences (AT-rich regions) or structures (G-quadruplex). ERp57/PDIA3 participates in gene expression (e.g., DKC1; MCOLN3), DNA repair, and transcriptional factor reduction. Endoplasmic reticulum: ERp57/PDIA3 promotes oxidative folding of glycoproteins in concert with lectin chaperones calnexin and calreticulin and is involved in retrotranslocation to cytosol of toxin, virus, and receptor. ER–mitochondrion: ERp57/PDIA3 is present in mitochondria-associated membranes (MAM), the regions of ER that are closely associated with mitochondria; ERp57/PDIA3 complexed with μ-calpain is able to cleave apoptosis-inducing factor (AIF) and interferes with the mitochondrial bioenergetic function via the inhibition of STAT3 Ser727 phosphorylation

In light of the above provided information about the involvement of ERp57/PDIA3 in a broad spectrum of physiological events (Fig. 1) and related diseases, the identification of inhibitors or activators specific for this protein is very important. Specific inhibitors of ERp57/PDIA3 could be used to better identify its mechanism of action and its role in physiological or pathological conditions. At the same time, where ERp57/PDIA3 is identified as a therapeutic target, the use of specific ERp57/PDIA3 inhibitors or activators could represent a possible tool for future therapeutic strategies.

## Abbreviations

1α,25(OH)2D3: 1α,25-Dihydroxyvitamin D_3_, biologically active form of vitamin D_3_
1,25D3-MARRS: 1α,25(OH)_2_D_3_ membrane-associated, rapid-response steroid-binding
4E‑BP1: Eukaryotic translation initiation factor 4E-binding protein 1
A549: Human lung cell line
AD: Alzheimer’s disease
AHR: Airway hyperresponsiveness
ALS: Amyotrophic lateral sclerosis
Bak: BCL2 antagonist/killer
BMP2: Bone morphogenetic protein-2
CALR: Calreticulin
CaMKII: Ca^2+^/calmodulin-dependent protein kinase II
ccRCC: Clear cell renal cell carcinoma
CMECs: Immortalized human cerebral microvascular cells
c-Src: Rous sarcoma oncogene
DBP: Vitamin D_3_ binding protein
DKC1: Dyskerin pseudouridine synthase 1
ER: Endoplasmic reticulum
Ev: Everolimus
GAMs: Glioma-associated microglia/macrophages
GB: Glioblastoma
HA: Hemagglutinin
HCC: Hepatocellular carcinoma
HeLa: Cervix adenocarcinoma cells
HepG2: Hepatocellular carcinoma cells
HUVECs: Human umbilical vein endothelial cells
IAV: Influenza A virus
ICD: Immunogenic cell death
IL-24: Interleukin-24
ILF3: Interleukin enhancer-binding factor 3
JNK: Jun amino-terminal kinases
Jurkat: Acute T-cell leukemia
KDELR1: KDEL endoplasmic reticulum protein retention receptor 1
LIF: Leukemia inhibitory factor
MAM: Mitochondria-associated ER membrane
MAPK: Mitogen-activated protein kinase
MCOLN3: Mucolipin 3
MDA-MB-468: Breast adenocarcinoma cells
MDCK: Madin–Darby canine kidney
MHC-I: Major histocompatibility complex I
MPL: Myeloproliferative leukemia protein
mTORC1: Mechanistic target of rapamycin complex 1
OGDR: Oxygen glucose deprivation and re-oxygenation
*P4HB*: Prolyl 4-hydroxylase subunit beta gene
PCa: Prostate cancer
PDIs: Protein disulfide isomerases
PERK: Protein kinase RNA-like ER kinase
PKC: Protein kinase C
PLAA: Phospholipase A2-activating protein
PLA2: Phospholipase A2
PrP: Prion protein
Ref-1/APE: Redox-factor 1/apurinic (apyrimidinic) endonuclease
SERCA: Sarcoendoplasmic reticulum (SR) Ca^2+^ transport ATPase
SAPK: Stress-activated protein kinases
STAT3: Signaling and transcription factors 3
TAM: Tumor-associated macrophages
TBI: Traumatic brain injury
TDP-43: TAR DNA-binding protein-43
TG2: Human transglutaminase 2
TMP: Tetramethylpyrazine
TNBC: Triple-negative breast cancer
TR: Thioredoxin
UPR: Unfolded protein response
VDR: Vitamin D receptor
WNT5A: Wnt family member 5A
ZP3: Glycoprotein that participates in ZP receptor complex
